# Small‐scale genetic structure and mating patterns in an extensive sessile oak forest (*Quercus petraea* (Matt.) Liebl.)

**DOI:** 10.1002/ece3.7613

**Published:** 2021-05-13

**Authors:** Pascal Eusemann, Heike Liesebach

**Affiliations:** ^1^ Thuenen‐Institute of Forest Genetics Großhansdorf Germany

**Keywords:** dispersal distances, forest trees, gene flow, genetic structure, mating patterns, reproduction dynamics

## Abstract

Oaks (*Quercus*) are major components of temperate forest ecosystems in the Northern Hemisphere where they form intermediate or climax communities. Sessile oak (*Quercus petraea*) forests represent the climax vegetation in eastern Germany and western Poland. Here, sessile oak forms pure stands or occurs intermixed with Scots Pine (*Pinus sylvestris*). A large body of research is available on gene flow, reproduction dynamics, and genetic structure in fragmented landscapes and mixed populations. At the same time, our knowledge regarding large, contiguous, and monospecific populations is considerably less well developed. Our study is an attempt to further develop our understanding of the reproduction ecology of sessile oak as an ecologically and economically important forest tree by analyzing mating patterns and genetic structure within adult trees and seedlings originating from one or two reproduction events in an extensive, naturally regenerating sessile oak forest. We detected positive spatial genetic structure up to 30 meters between adult trees and up to 40 meters between seedlings. Seed dispersal distances averaged 8.4 meters. Pollen dispersal distances averaged 22.6 meters. In both cases, the largest proportion of the dispersal occurred over short distances. Dispersal over longer distances was more common for pollen but also appeared regularly for seeds. The reproductive success of individual trees was highly skewed. Only 41 percent of all adult trees produced any offspring while the majority did not participate in reproduction. Among those trees that contributed to the analyzed seedling sample, 80 percent contributed 1–3 gametes. Only 20 percent of all parent trees contributed four or more gametes. However, these relatively few most fertile trees contributed 51 percent of all gametes within the seedling sample. Vitality and growth differed significantly between reproducing and nonreproducing adult trees with reproducing trees being more vital and vigorous than nonreproducing individuals. Our study demonstrates that extensive, apparently homogenous oak forests are far from uniform on the genetic level. On the contrary, they form highly complex mosaics of remarkably small local neighborhoods. This counterbalances the levelling effect of long‐distance dispersal and may increase the species’ adaptive potential. Incorporating these dynamics in the management, conservation, and restoration of oak forests can support the conservation of forest genetic diversity and assist those forests in coping with environmental change.

## Graphical abstract

Extensive, apparently homogenous oak forests are far from uniform on the genetic level. On the contrary, they form highly complex mosaics of remarkably small local neighborhoods. This counterbalances the levelling effect of long‐distance dispersal and may increase the species’ adaptive potential. Incorporating these dynamics into the management, conservation, and restoration of oak forests can support the conservation of forest genetic diversity and assist those forests in coping with environmental change.

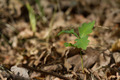

## 1 INTRODUCTION

Oaks (*Quercus*) are major components of temperate forest ecosystems in the Northern Hemisphere where they form intermediate or climax communities. So far‐reaching is the ecological importance of the genus, that oaks were called “the most important woody genus in the Northern Hemisphere” (Nixon, 1997). Due to their ecological dominance and their species richness, oaks are an important model clade for the study of the ecology and evolution of long‐lived, ecosystem‐defining plants (Cavender‐Bares, 2019).

In addition to their ecological importance, some oak species are also of considerable economic value. The continued use of oaks over centuries, together with the transformation of forests into farmland, has substantially altered the structure and distribution of oak forests, sometimes leading to a drastic decrease in forest cover while simultaneously increasing fragmentation of the remaining forests (Bunce, 1982; Ellenberg, 2009; Packard & Mutel, 1997; Timbal & Aussenac, 1996).

Aided by the advance of molecular methods, the last two decades have seen an immense progress in research regarding the effects of fragmentation, isolation, population structure, successional stage or environmental conditions on gene flow, reproduction dynamics, and genetic structure in oak populations. Across species and ecological settings, these studies uncovered remarkable long‐distance dispersal capabilities for both pollen (Buschbom et al., 2012; Gerber et al., 2014) and seeds (Hosius et al., 2012) and demonstrated that fragmented stands are usually much better connected by gene flow than would be expected by their physical appearance. At the same time, it became apparent that the interplay between long‐ and short‐distance dispersal is highly dynamic and depends strongly on population structure (Pakkad et al., 2008). Gene flow can be surprisingly limited in stands of higher density (Chybicki & Burczyk, 2010; Nakanishi et al., 2009). This dynamic is biologically beneficial by ensuring genetic exchange in sparsely populated areas while enabling local adaptation in large populations with high density (Chybicki & Burczyk, 2010; Fernández‐Manjaréz et al., 2006).

A pronounced disproportion in individual reproductive success among the individuals of a population also appears to be a universal phenomenon (Chybicki & Burczyk, 2013). All studies that addressed this issue revealed that only a minority of all individuals actually participate in reproduction. Among the reproductively active trees, usually only a handful of individuals provide the majority of all offspring (Gerber et al., 2014; Gerzabek et al., 2017; Lepais & Gerber, 2011; Moracho et al., 2018; Moran & Clark, 2012). While the majority of these studies analyzed one single reproductive event, Lepais and Gerber (2011) and Moran and Clark (2012) studied seedlings aged one to twelve years. These represent several reproductive cycles and indicate that heterochronic masting alone, while most likely an important factor, may not be sufficient to explain the observed unbalanced parental contributions.

Sessile oak (*Quercus petraea* (matt.) liebl.) forests represent the climax vegetation in eastern Germany and western Poland (Bohn & Neuhäusl, 2000; Ellenberg, 2009; Hofmann & Pommer, 2004). Here, sessile oak forms pure stands or occurs intermixed with Scots Pine (*Pinus sylvestris* L.). Despite a long tradition of silvicultural use, extensive stands considered to represent the natural state of this vegetation community can still be found in the region (Bohn & Neuhäusl, 2000).

Throughout its pan‐European distribution range, the structure of sessile oak populations ranges from scattered, fragmented, and heavily altered stands (Buschbom et al., 2012; Timbal & Aussenac, 1996; Vranckx, Mergeay, et al., 2014) to large, contiguous stands (Gerber et al., 2014). The species often grows mixed with other interfertile oak species (Chybicki & Burczyk, 2010; Gerber et al., 2014; Streiff et al., 1999). Consequently, previous studies focused on a wide range of population structure: stands in fragmented landscapes (Vranckx, Jacquemyn, et al., 2014; Vranckx, Mergeay, et al., 2014), stands at the species’ distribution limits (Muir et al., 2004; Valbuena‐Carabaña et al., 2005), or stands consisting of several interfertile oak species (Chybicki & Burczyk, 2010; Streiff et al., 1999). Therefore, a large body of research is available on gene flow, reproduction dynamics, and genetic structure in a wide range of different ecological settings. At the same time, our knowledge regarding large, contiguous populations is considerably less well developed and those studies that addressed these phenomena in more extensive forests mostly did so in mixed stands (Chybicki & Burczyk, 2010; Gerber et al., 2014; Streiff et al., 1999). In order to complete our understanding of the processes that ultimately drive adaptation and evolution in sessile oak, we therefore took the opportunity to study gene flow, reproduction, and genetic structure in an extensive near‐natural stand composed exclusively of sessile oak.

Gaining insights into the dynamics in forests considered to represent the natural state of their respective vegetation community is important, even if such stands now only form a minority of sessile oak stands throughout Europe. The genetic consequences of different forest management practices (Finkeldey & Ziehe, 2004; Ratnam et al., 2014) and the importance of managing forest genetic resources (Loo et al., 2014; Potter et al., 2017) have gained increasing attention during the last years and forest management practices emulating natural processes are regularly suggested as appropriate tools to address these issues (Jõgiste et al., 2017; Remeš, 2018; Spathelf et al., 2018). To succeed in this, a comprehensive understanding of these processes in natural and near‐natural populations is needed.

Our study is an attempt to further develop our knowledge of the reproduction ecology of sessile oak as an ecologically and economically important forest tree and provide insights that may assist in the management and regeneration of production forests as well as in the restoration of degraded populations. In order to achieve this, we studied reproduction and dispersal patterns in *Q. petraea* and compared genetic diversity and spatial genetic structure between adult trees and naturally established seedlings, focusing on (1) seed and pollen dispersal distances, (2) distribution of individual reproductive success, and (3) spatial genetic structure and genetic diversity within and across both generations.

## 2 MATERIAL AND METHODS

### 2.1 Study area and plant material

The study site is located within the “Schlaubetaler Eichen” oak forest in eastern Brandenburg, Germany (52°10’15’’ N, 14°28’32’’ O, Appendix S1). Covering an area of 614 hectares, this forest represents the largest contiguous sessile oak population in northeastern Germany. Here, forests dominated by *Q. petraea*, locally in association with Scots pine, naturally form the climax vegetation (Bohn & Neuhäusl, 2000; Ellenberg, 2009; Hofmann & Pommer, 2004). The particular locality of our study is considered to represent a characteristic example of the forest type in its natural state (Bohn & Neuhäusl, 2000). Here, the forest is characterized by a single, closed‐canopy layer and dense ground cover vegetation. The tree layer is composed exclusively of sessile oak with a density of about 246 trees/ha. As an approved seed stand, the stand is currently managed for seed production but not for timber production. The age of the stand is about 150 years (Ziesche et al., 2013). The oldest trees in the area are aged up to 300 years. No obvious, pronounced age structure, for example, strong variation in diameter, is visible at our particular study site. Reproduction in oak starts at an age of about 20 years. All trees within the tree layer can therefore be regarded to be of reproductive age. Unfortunately, no individual age data are available for trees within the adult generation and no detailed knowledge about the age range within the tree layer is available. Without further knowledge about the specific history of the stand, it is therefore possible that the current tree layer comprises trees of two or three successive generations.

The understory vegetation is characterized by *Calamagrostis arundinacea* (l.) roth (Poaceae) and *Vaccinium myrtillus* L. (Ericaceae). Browsing pressure by deer is high and oak seedlings can be found mostly in fenced areas, where herbivore access is prevented. As the forest is not managed for timber production at our study site, regeneration sites suitable for long‐term establishment of seedlings are therefore restricted to crown openings caused by windthrow and other natural causes. As no such sites are present in our particular study plot, there is no subcanopy vegetation of older oak saplings and the forest is strictly divided into the canopy layer formed by adult trees and the understory formed by grasses, blueberry, and young oak seedlings. The adult tree and seedling cohorts therefore represent two discreet generations without overlap. Plants within the seedling generation uniformly measure about 40 cm in height and most likely originate from only one or two reproduction events. Long‐term establishment of the light‐demanding seedlings is not possible in the absence of suitable regeneration sites caused by crown openings and no larger and obviously older saplings are present within the study site.

**FIGURE 1:**
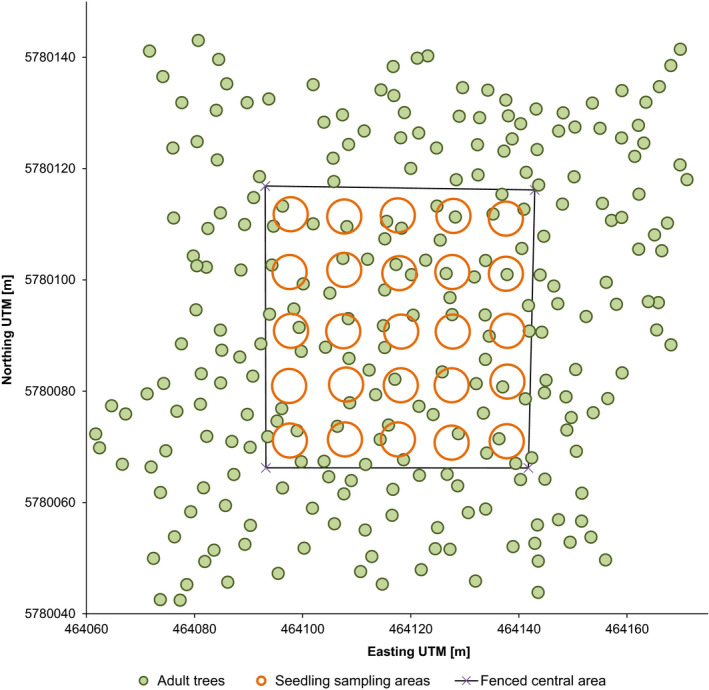
Map of the study site. Seedling sampling areas indicate both location and extent of the sampling areas

To address our questions, we used an already established long‐term monitoring plot with an extension of 100 x 100 meters (1.0 ha, Figure 1). In the center of the plot, an area of 50 x 50 meters (0.25 ha) is fenced in to exclude large herbivores and therefore to enable natural regeneration undisturbed by herbivore browsing. Within the one‐hectare plot, all adult trees had previously been marked, mapped, and their social status determined according to the Kraft scale. The Kraft scale is used to classify the vitality and vigor of individual trees in a population by estimating tree height and crown development in relation to all other trees in the stand (Kramer, 1988). The index includes five stages: social class 1 (predominant trees with exceptionally well‐developed crowns, potentially overtopping the main canopy layer), class 2 (dominant trees with well‐developed crowns), class 3 (co‐dominant trees with normally developed crowns, but crown development is restricted by neighboring trees), class 4 (dominated, partially overtopped trees), and class 5 (suppressed, entirely overtopped trees).

These data were provided by the Landeskompetenzzentrum Forst Eberswalde. DNA samples were collected from all living adult trees. As DNA samples, we used leaf material if possible. When leaves were out of reach from the forest floor, cambium samples were taken instead. Naturally regenerated seedlings were sampled within the fenced core area by establishing a grid of 25 evenly distributed, circular sampling sites with a diameter of six meters (Figure 1). We collected leaf samples of 20 seedlings per site. In total, we sampled all 246 adult trees within the stand and 500 seedlings.

### 2.2 Genotyping

DNA was extracted from leaf and cambium samples following the protocol of Dumolin et al. (1995). Genotyping was performed using 19 microsatellite markers combined into two multiplex assays developed by Guichoux et al. (2011). Though representing mostly dinucleotide motifs, this marker set was developed with a particular focus on genotyping accuracy. To achieve this, low‐quality markers suffering from null alleles, stutter bands, triple bands, unspecific products, and weak or heterogeneous amplification were redesigned or excluded from the initial set of more than 70 candidate markers (Guichoux et al., 2011). To verify marker quality, we performed initial tests using replicate amplifications of a sample of 24 oaks. The set mostly proved to produce reproducible and unambiguous profiles. Only marker PIE152 performed poorly in our samples and was therefore excluded from the original set of 20 markers. PCRs were done using the Qiagen Multiplex PCR kit following manufacturer instructions (Qiagen, Hilden, Germany). Separation and analysis of PCR products were done on a CEQ 8,000 capillary sequencer (Beckman Coulter, Framingham, MA, USA). Fragment analysis and genotyping were performed using GenomeLab software 10.2.3 (Sciex, Framingham, MA, USA). To ensure accurate and consistent genotyping, all individual profiles were manually checked and verified. This was done by the same editor for the whole dataset in order to avoid introducing a human "disagreement error” (Guichoux et al., 2011). Additionally, all profiles expressing alleles that occurred only once in the entire dataset were revisited to ensure correct allele calling.

### 2.3 Data analysis

#### 2.3.1 Parentage reconstruction

We used the full‐likelihood approach to parentage analysis implemented in the software COLONY 2.0.6.4 (Jones & Wang, 2010) to identify parental relationships between adult trees and seedlings and to reconstruct the number and genotypes of additional, unsampled parents. The initial values for null allele frequencies for each population were calculated with Cervus (Kalinowski et al., 2007). Parentage analyses were repeated twice with differing initial random seeds, resulting in an error rate of 1.4 percent and thereby confirming that both genetic structure within the sample and marker quality were sufficient to produce reliable results. Only parent–offspring and sibship relations with a probability above 0.95 were included in subsequent analyses. Detailed parameter settings used for the parentage analysis are shown in Appendix S2.

We used seedling and adult tree spatial positions and parent–offspring relationships to calculate pollen and seed dispersal distances. Seed dispersal distances were calculated as the Euclidean distance between a seedling and its seed parent. Accordingly, pollen dispersal distances were calculated as the distance between the two parents of a given seedling. If two parents were identified for a seedling, we classified the nearest parent as the seed parent. If only one parent was identified, we classified it as the seed parent. This approach is especially suited to oak species considering the heavy seeds, the pollination by wind and the large difference in seed and pollen weight. This assumption is also in agreement with studies on the behavior of animal dispersers in sessile and pedunculate oak. While rodents act as short‐distance dispersers usually over distances of up to ten meters (Bogdziewicz et al., 2019, den Ouden et al., 2005), jays usually disperse seeds over distances of several hundred meters (den Ouden et al., 2005). A few erroneous seed and pollen parent identifications introduced by dispersal events outside these typical animal dispersal distances cannot be ruled out but are not expected to introduce a systematic bias. Therefore, on the spatial extent of our study site (100x100m), this assignment scheme is considered to produce reliable parent identification and has been implemented with success in previous studies on oaks and other tree species (Bacles et al., 2006; Dow & Ashley, 1996; Gallagher et al., 2013; Gerber et al., 2014; Nakanishi et al., 2009; Oddou‐Muratorio & Klein, 2008).

In addition to parentage analysis in two‐generation samples, COLONY is also able to infer sibship relations in samples without parental information (Jones & Wang, 2010; Wang, 2004; Wang & Santure, 2009). We used the full‐likelihood approach implemented in the software to identify individuals in full‐ and half‐sibship relations within the adult generation in order to assess the number and size of families within the adult tree population. As we cannot rule out that the adult generation represents individuals of more than one single generation, the inferred full‐sib relationships possibly contain pairs of individuals in all first‐degree relationships, that is, full‐sibs as well as parent–offspring. Accordingly, the inferred half‐sib relationships might include pairs of individuals in all second‐degree relationships, that is, half‐sibs, grandparent–grandoffspring, and “uncle/aunt”–“nephew/niece.” Individual tree age data would allow to assess whether the adult generation represents a multi‐generation population. Unfortunately, such data were not available for the study population. Therefore, the inferred families must be regarded as closely related individuals in a number of different first‐ and second‐degree relationships. We then used the spatial positions of the adult trees to visualize the spatial extent and distribution of these families within the stand. Detailed parameter settings for the adult generation sibship analysis are shown in Appendix S2.

#### 2.3.2 Genetic diversity

The ability of the marker set to reliably identify individuals was verified by calculating probabilities of identity (P_ID_) on both the population and the full‐sibling level (Peakall et al., 2006; Taberlet & Luikart, 1999; Waits et al., 2001). To assess the genetic diversity of the adult and offspring generation, we calculated absolute (*A*) and effective number of alleles (*A*_E_), private alleles (*A*_P_), observed (*H*_O_) and expected (*H*_E_) heterozygosity, and effective population size (*N*_E_). Except for effective population size, all calculations were performed using GenAlEx 6.503 (Peakall & Smouse, 2006, 2012).

To estimate effective population size, we employed the marker‐based single sample approach implemented in COLONY 2.0.6.4 (Wang, 2004). Calculation of *N*_E_ was carried out separately for the adult and the seedling generation. In addition, we calculated a demographic *N*_E_ using the classic formula *N*_E_=4N/(2+Variance *k*), derived by Wright (1938) for randomly mating populations of hermaphroditic diploid organisms. The number of gametes *k* that contributed to each generation was estimated by the COLONY analyses, considering only parent pairs with *p* > .95. For the adult generation, estimation of *k* relied on reconstructed parents. For the seedling generation, *k* was estimated based on parents identified among the sampled adult trees and reconstruction of additional, unsampled parents. In both cases, *k* therefore does not correspond to the total number of gametes produced by a parent tree, but to the number of gametes that contributed to the analyzed sample of adult trees and seedlings. As the parentage analysis is based on a systematic sampling of adult trees and seedlings, both the number of gametes *k* and the number of offspring attributed to an adult tree represents not the absolute, but the relative reproductive success (Truffaut et al., 2017).

#### 2.3.3 Spatial genetic structure (SGS) and genetic differentiation

To assess spatial genetic structure within the adult and the seedling generation, we calculated Moran's I using Hardy and Vekemans’ (1999) pairwise genetic relationship coefficient and averaging it over distance classes of 10 meters for all analyses to obtain mean values of Moran's I (Dewey & Heywood, 1988). Statistical significance was established by random permutation of spatial positions of all individuals with 1,000 iterations. All SGS calculations were performed using SpaGeDi 1.5 (Hardy & Vekemans, 2002).

We used AMOVA to assess the partitioning of genetic diversity between the two generations as well as between the 25 seedling sampling sites. Statistical significance was tested using random permutation with 1,000 iterations. Genetic differentiation between the two generations and between the 25 seedling sampling sites was estimated by calculating Wright's fixation index *F*_ST_ and Jost's estimator of differentiation *D*_EST_ (Jost, 2008). Wright's *F*_ST_ is based on analysis of variance of allele frequencies and is influenced by heterozygosity. Jost's *D*_EST_ on the other hand is based on the effective number of alleles and is unaffected by population size. Statistical significance for differentiation analyses was established by bootstrapping with 1,000 iterations. AMOVA as well as *F*_ST_ and *D*_EST_ estimations were performed using GenAlEx 6.503 (Peakall & Smouse, 2006, 2012).

## 3 RESULTS

The one‐hectare study site contained 246 adult trees. Twenty seedlings were collected per sampling point (Figure 1). Some seedlings were accidentally sampled twice. This was caused by undetected branching of some seedlings within the strongly developed cover of *Vaccinium myrtillus*. Therefore, the 500 collected samples were reduced to a final total of 487 seedlings. The final number of seedlings per sampling site ranged from 18 to 20.

The overall probability of identity (*P*_ID_) of the marker set was 1.4 × 10^‐22^. The P_ID_ of full siblings was 2.1 × 10^‐8^. This resolution allows unambiguous identification of individual genotypes. Identical genotypes can therefore safely be assigned to accidental duplicate sampling and do not indicate insufficient genotyping resolution.

### 3.1 Genetic diversity

The genetic diversity parameters were almost identical within the adult and offspring generations (Table 1). The absolute number of alleles per locus ranged from 7 to 25 in the adult and from 6 to 23 in the seedling generation. The effective number of alleles amounted to about one third of the absolute number of alleles in both generations. Most alleles were rare, 69 percent of all alleles occurred with a frequency <0.05 and 35 percent with a frequency <0.01. Fourteen percent of all alleles occurred in only one single individual.

**TABLE 1:** Population genetic parameters of the adult and seedling cohort within the study stand

	*N*	*A*	*A*_E_	*A*_P_	*H*_O_	*H*_E_	*F*	*N*_E_		
								rand	non	dem
Adults	246	14.37 (±1.33)	5.54 (±0.76)	1.26 (±1.24)	0.73 (±0.04)	0.75 (±0.04)	0.04 (±0.02)	169 (136/214)	164 (129/207)	190
Seedlings	487	14.42 (±1.22)	5.34 (±0.72)	1.32 (±1.00)	0.72 (±0.04)	0.74 (±0.04)	0.05 (±0.04)	173 (141/214)	166 (134/211)	196

Abbreviations: *A*, number of alleles (in brackets: standard error); *A*_E_, effective number of alleles; *A*_P_, number of private alleles; dem, demographic model; *F*, fixation index; *H*_E_, expected heterozygosity; *H*_O_, observed heterozygosity; *N*, number of individuals; *N*_E_, effective population size (in brackets: upper/lower 95% confidence intervals); non, nonrandom mating model; rand, random mating model.

Both generations showed a comparable number of private alleles. This was also reflected by the total number of alleles of the whole dataset (adults and seedlings combined) which reached 8 to 27 alleles per locus and exceeded the number of alleles in any single generation.

The genetic homogeneity of the two generations was further illustrated by the results of the AMOVA: Only 0.2 percent of the genetic diversity was partitioned among the two generations. As expected in a large, naturally regenerating population, the majority of the genetic diversity (93.8 percent) was partitioned within both generations. Still, a comparatively large proportion of 6.0 percent was partitioned within individuals.

The estimated effective population size was comparable for all statistical models applied (marker based under assumption of random and nonrandom mating, demographic approach) as well as between the two generations (Table 1). N_E_ amounted to about 71 percent of the census size in the adult generation and about 72 percent in the seedling generation.

### 3.2 Spatial genetic structure and genetic differentiation

**FIGURE 2:**
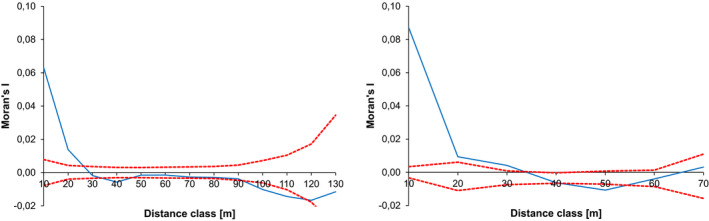
Spatial genetic structure within the study stand. Left: between adult trees. Right: between seedlings. Solid blue line: estimated Moran's I, dotted red lines: upper and lower 95% CI

Significant spatial genetic (SGS) structure was detected within the adult generation as well as the seedling generation (Figure 2). Positive correlation indicates local clusters of relatives and allows estimating their size. Significant positive SGS was detected up to 20–30 meters between adult trees and up to 30–40 meters between seedlings.

Differentiation between the adult and seedling generation was very low and nonsignificant (*F*_ST_=0.001, *D*_EST_=0.003, *p* > .05). There was pronounced genetic differentiation in both estimators between most of the seedling sampling areas (Appendix S3): *F*_ST_ averaged 0.05 between sampling areas (range 0.00–0.12) and *D*_EST_ averaged 0.12 (range 0.00–0.27). To test for significant differentiation, p‐values were Bonferroni‐corrected for multiple testing. The genetic differentiation between seedling sampling areas was significant in 87.3 percent of all cases for F_ST_ and in 89.7 percent for D_EST_ after correction (*p* < .002).

### 3.3 Reproduction dynamics

We identified a total of 181 families representing full‐ and half‐sib relationships within the adult generation. Family sizes ranged from one to 13 members. The majority of these (81 percent) contained 1–3 members. Only 19 percent of all families included more than three members. However, due to their larger sizes, these families represented a total of 40 percent of all individuals in the adult generation. Appendix S4 illustrates the family structure and gives an impression on the spatial distribution and extent of families within the stand.

Within the seedling generation, both parents could be identified within the sampled adult generation for 24 percent of all seedlings. No case of selfing was observed. For 60 percent of all seedlings, only one parent could be identified within the stand. For 16 percent of the seedlings, no parent could be identified within the stand.

**FIGURE 3:**
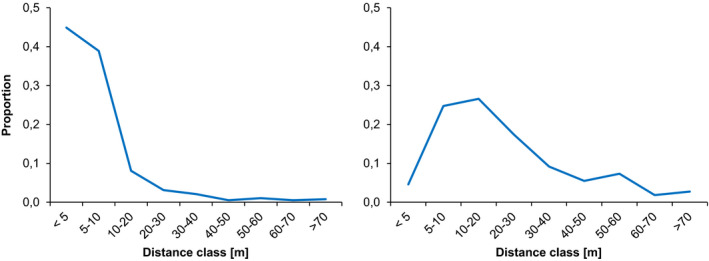
Dispersal distances of seed (left) and pollen (right)

Seed and pollen dispersal distances are shown in Figure 3. In both cases, the largest proportion of the dispersal occurs over very short distances. Seed dispersal averages 8.4 meters (±10.8 meters, median 5.4 meters, range 0.2–81.9 meters). Pollen dispersal averages 22.6 meters (±17.4 meters, median 16.7 meters, range 3.2–72.8 meters).

**FIGURE 4:**
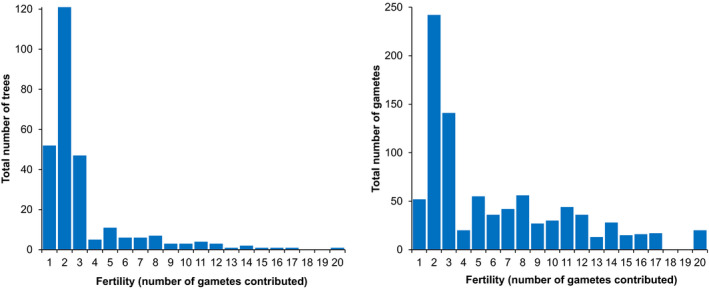
Distribution of individual fertility of trees participating in reproduction (left) and total number of gametes contributed per fertility category (right). The identification of trees contributing to the seedling cohort is based on sampled adult trees and reconstruction of additional, unsampled trees. The total number of trees participating in reproduction is therefore larger than the sampled adult cohort

The reproductive success of individual trees was highly skewed (Figure 4). Of the 246 adult trees in the stand, only 41 percent were identified as parents among the 487 seedlings. With 59 percent, the majority of adult trees did not appear as parents within the seedling sample. Among those trees that contributed to the seedling sample, 80 percent contributed 1–3 gametes. Only 20 percent of all parent trees contributed four or more gametes. However, these relatively few most fertile trees contributed 51 percent of all gametes within the seedling sample.

**FIGURE 5:**
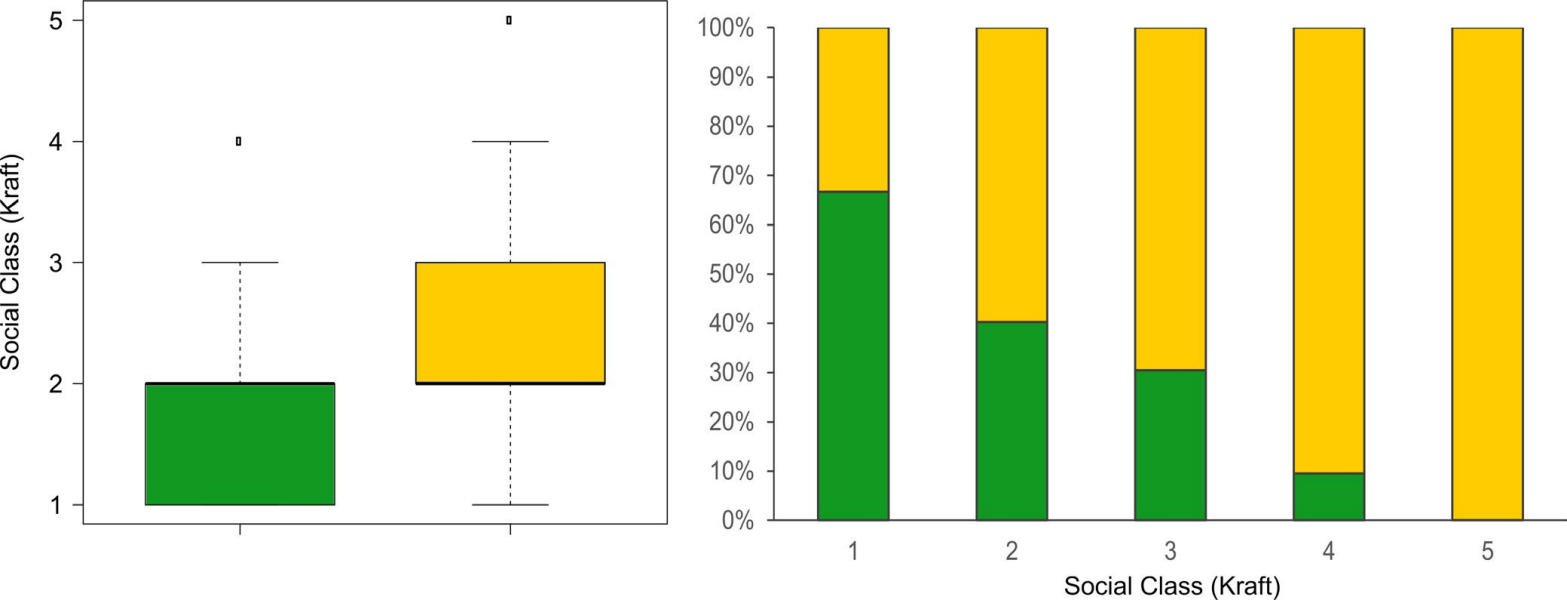
Distribution of reproductively active/inactive trees over social classes (left). Proportion of reproductively active/inactive trees in each social class within the study population (right). Dark green: Reproducing trees. Yellow: Nonreproducing trees

Reproducing and nonreproducing trees differed strongly in their social class (Figure 5). The mean social class was 1.9 in reproducing trees and 2.5 in nonreproducing trees. The difference in social class between reproducing and nonreproducing trees was highly significant (Mann–Whitney U test, U = 5,308, *p* = 6.5 × 10^‐7^). The effect size after Cohen (1992) was *d* = 0.683, indicating an effect of medium strength. The proportion of reproducing trees showed a constant decline from a maximum of 67 percent in social class 1 (predominant trees with exceptionally well‐developed crowns) to 10 percent in class 4 (dominated, partially overtopped trees). No tree in class 5 (suppressed, entirely overtopped trees) participated in reproduction.

## 4 DISCUSSION

Our goal was to characterize the mating system of sessile oak in extensive, naturally regenerating forests. As would be expected in a stable, large, and uninterrupted population comprising tens of thousands of individuals, the genetic diversity of the adult population was preserved within the next generation and we observed no differences in the genetic structure or effective population size between the two generations. Changes in *N*_E_ between generations indicate changes in the underlying mating system, gene flow, selection, genetic drift, or inbreeding rate (Wang et al., 2016). The genetic homogeneity, preservation of the genetic diversity, and comparable *N*_E_ between the two generations support the assumption of near‐naturalness and long‐term stability in the regeneration of the forest at the study site.

Rare alleles play an important role in the genetic structuring of the study population both in the adult and the seedling generation: two thirds of all alleles occur with a frequency of less than five percent and one third with less than one percent. This is also visible in the small effective number of alleles compared with the absolute number of alleles. The effective number of alleles reflects the allele frequency distribution of common alleles, while rare alleles have a negligible influence. The low effective number of alleles therefore indicates a small number of common alleles and a large number of rare alleles in the study population. The impact of rare alleles on the genetic structure of the study population is also shown by the AMOVA: While genetic diversity was not partitioned between the two generations, a surprisingly large proportion of it was partitioned within individuals. This is explained by the occurrence of rare alleles that appear only in single individuals, thus creating pronounced individual‐based genetic structure.

The occurrence and accumulation as well as the mendelian transmission of intraindividual somatic mutations in long‐lived forest trees have recently been described for European oaks (Plomion et al., 2018; Schmid‐Siegert et al., 2017) and Sitka spruce (Hanlon et al., 2019). Both Plomion et al. (2018) and Hanlon et al. (2019) argued that the accumulation of somatic mutations in long‐lived trees might increase genetic diversity and lead to an accumulation of rare alleles within a population over time. We observed such an accumulation in our study population of sessile oak and the observations of Plomion et al. (2018) and Hanlon et al. (2019) might explain the underlying mechanism that leads to this phenomenon. Following this line of thought, the question arises whether high rates of rare alleles in populations of forest trees point toward old stands that were able to regenerate over several generations without large‐scale disturbances and therefore accumulated high rates of rare alleles over a long period of time. Unfortunately, very few studies so far have analyzed the distribution of rare and common alleles within populations, making it difficult to test this assumption or to place our results into a wider population genetic context. However, the few data available (Mariette et al., 2002) indicate that the proportions of rare and common alleles found in our study population are in fact representative for large, old, naturally established forests of sessile and pedunculate (*Q. robur* L.) oak in Europe.

Dispersal over shortest distances prevails in both pollen and seed. As expected for a wind‐pollinated species with heavy seeds, dispersal over longer distances is more common for pollen but also occurs regularly for seeds. It is important to call to mind that our study stand is not an isolated unit, but is embedded within an extensive population. Our plot boundaries do not mark physical boundaries and the forest expands beyond them uninterrupted and identical in structure and density in each direction. Looking at the dimensions of the study stand (Figure 1), it becomes apparent that all seedling sampling areas are within reach for seeds originating from trees outside the plot boundaries. It is therefore not surprising that a quarter of all seedlings originated from trees outside the stand. The same holds true for the large proportion of pollen from outside the stand. Given the pronounced tail of the pollen dispersal curve of up to more than 70 m, it follows that each individual tree is likely to be pollinated by trees from a total area of up to 2 hectares, reaching well beyond the limits of our study site even for individuals within the core area where the seedling sampling was performed. Considering the homogenous structure of the forest and a density of 246 trees/ha, this represents an estimated total of approximately 500 trees that act as potential pollen donors for each individual tree.

Gleaves (1973) postulated that dispersal distances in pollen should depend on population density. And in fact, a strong dependency of pollen dispersal distances on population density and size has been shown in various oak species (Pakkad et al., 2008; Vranckx, Jacquemyn, et al., 2014; Vranckx, Mergeay, et al., 2014). In our study, this principle is brought to an extreme by the very high population density of 246 trees/ha. To our knowledge, comparably small pollen dispersal distances in oaks have only been reported by Moracho et al. (2016) in small stands of *Q. robur* in an otherwise strongly fragmented population and by Smouse et al. (2012) in *Q. alba* L. forests with a density of 93 trees/ha. To a lesser degree, seed dispersal distances also seem to be affected by population density. With a mean of 8.4 meters, seed dispersal distances in our study population are among the lowest reported so far. Chybicki and Burczyk (2010) reported dispersal distances of 8.8 and 15.6 meters in two mixed stands of *Q. petraea* and *Q. robur*. Nakanishi et al. (2009) observed mean dispersal distances of 16.8 meters for *Q. salicina* Blume, also in a naturally regenerated high‐density population.

Remarkably long‐distance dispersal events have been documented both for pollen (Buschbom et al., 2011; Gerber et al., 2014) and seed (Hosius et al., 2012) in oaks. In our study population, dispersal curves for both pollen and seed show a clear maximum at very short distances, followed by a pronounced drop in dispersal events (Figure 3). This tail is naturally broader for pollen than for seed but shows a steady decline over distance in both cases. The small local neighborhoods created by such limited dispersal create “opportunities for local selection and drift” (Fernández‐Manjaréz et al., 2006) in extensive populations of species with strong dispersal capabilities and hence may play an important role for the adaptive potential and evolution of such species.

These restricted local neighborhoods also become visible in the pronounced spatial genetic structure of the seedling generation. Positive spatial genetic structure almost identical in scale to our results has also been detected in populations of *Q. robur* (Hampe et al., 2010) and *Q. salicina* (Nakanishi et al., 2009). In addition to this, we were able to demonstrate that spatial genetic structures exist on a scale even below the one detected by the SGS analysis. Under the conditions of our study population, seed dispersal is limited enough to cause significant genetic differentiation between samples of 18–20 seedlings over distances as short as ten meters and creates a unique composition of parents within each of these samples (Figure 2 and Appendix S5). Mitton et al. (1989) and Mitton et al. (1998) observed similar within‐stand genetic differentiation over smallest distances at adaptive loci, providing strong evidence of the functional dimension of small local genetic neighborhoods and their importance for local adaptation.

Pronounced spatial genetic heterogeneity is not only found in the seedling generation, but also in the adult generation, which is characterized by a complex family structure consisting of numerous small, tightly clustered families (Figure 2 and Appendix S4). It has to be pointed out that while the study population is a single‐layer canopy forest, it cannot be ruled out that the adult generation actually consists of individuals spanning more than one single generation. Therefore, we have to take into consideration that our inferred families might represent individuals in a number of different relationships. Fortunately, this does not impair our conclusions. Our reconstruction of the adult generation family structure might slightly overestimate sibling‐family sizes when compared to the true sibling families within the seedling generation. But even in this case, the inferred families still represent core families consisting only of very closely related individuals.

Despite these possible limitations, the similarities in the genetic structure of the adult and the seedling generation are striking. The spatial extent of the positive spatial genetic correlation is almost identical (Figure 2). The slightly higher correlation and spatial extent in the seedling generation will possibly converge toward the pattern observed in the adult generation by future mortality of seedlings. Nakanishi et al. (2009) have compared spatial genetic structure in adults and seedlings in an unmanaged, naturally regenerated population of *Q. salicina* and also found comparable patterns in both generations. Family structures are also very similar in both generations with eighty percent of all individuals belonging to small families of up to three members and only a minority of twenty percent forming larger families of more than three members. However, in both generations these large families make up half of all individuals in the seedling generation and still forty percent in the adult generation. As with spatial genetic structure, future mortality of seedlings might lead to an even closer match of these figures in years to come. To our knowledge, no other study so far has attempted to resolve genetic structure in oak populations beyond the results of spatial genetic structure analyses. While plausible, it is therefore difficult to assess whether the family structure observed in our population is characteristic of old, naturally regenerating oak forests in general.

Highly unequal distribution of individual reproductive success (Figure 4) has been observed repeatedly and seems to be a characteristic phenomenon in populations of oaks and other forest tree species (Alexandre et al., 2020; Gerber et al., 2014; Lepais & Gerber, 2011; Moran & Clark, 2012; Truffaut et al., 2017). Gerzabek et al. (2017) hypothesized that strongly skewed individual reproductive success could be typical during the initial establishment phase of a population and will level out when population size and age increase. Our results from an old, large, and stable population show that inequalities in reproductive success apparently are not a function of a population's developmental stage. Our results indicate that the underlying mechanism causing this inequality might be found in individual tree growth.

We were able to establish that there is no long‐term trade‐off between reproduction and growth in our closed‐canopy population: the larger and more dominant trees get, the more likely they will participate in reproduction (Figure 5). This effect is pronounced enough, that even partial overtopping strongly limits an individual's reproductive success while completely overtopped individuals seem to drop out of the reproducing community entirely. This conclusion is backed up by Moracho et al. (2018) who found a connection between tree fertility and size as well as Alexandre et al. (2020) who observed strong variation in individual reproductive success and a clear relationship between reproductive success and growth. If individual tree size and social status (as a proxy for size and light exposure) are driving factors in determining individual reproductive success, the distribution of these two parameters among the individuals of a population will determine how pronounced the skew in individual reproductive success in this population will be. It remains to be studied how strong size and social status impact reproductive success under different ecological settings. It seems plausible to expect that the effect is more pronounced in closed‐canopy forests than in park‐like forests or savanna settings with reduced intraspecific competition. In our study population, one third of all trees within the highest social class did not participate in reproduction. Therefore, while social class gives an impression about which individuals within a stand are most likely to contribute to the next generation, it is not possible to identify reproductively successful trees with absolute certainty from this parameter alone.

Regarding the unequal distribution of individual reproductive success, it has to be noted that our results most likely reflect, to some degree, a sampling effect. We analyzed a sample of 487 seedlings from the center of the study site, most likely representing only one or two reproductive events. Nussbaumer et al. (2016) have demonstrated that within‐stand masting synchrony between individual trees is only weak in sessile oak. Gerber et al. (2014) have presented evidence of between‐year variation in individual reproductive success. While unbalanced parental contributions have been detected in many studies (Chybicki & Burczyk, 2013; Gerber et al., 2014; Gerzabek et al., 2017; Moracho et al., 2018; Moran & Clark, 2012; Truffaut et al., 2017), it can be assumed that the effect will be less pronounced if a larger sample involving more reproductive events is analyzed. And indeed, Truffault et al. (2017), who analyzed 2,510 seedlings representing a cumulation of 12 years of reproduction, found a much higher proportion of reproductive trees but the uneven distribution of individual reproductive success remained unchanged. Given the importance of individual reproductive success for adaptive and evolutionary processes, these results all serve to reinforce the conclusion of Gerber et al. (2014) that additional studies on a larger temporal and spatial scale are needed in order to shed more light on this phenomenon.

## 5 CONCLUSION

Additive genetic variation in adaptive traits is a prerequisite for selection and adaptation in forest trees (Myking, 2002). Genetic effects for both quality‐related and adaptive traits and significant family differences in such traits are well documented for several oak and other tree species (Adams et al., 2007; Baliuckas & Pliura, 2008; Berguson et al., 2017; Caignard et al., 2019; Kolb & Steiner, 1989; Kriebel, 1965, 1993; Kriebel et al., 1988; Míguez‐Soto & Fernández‐López, 2015; Pliura & Eriksson, 2002; Stoehr et al., 1998; Zhao et al., 2019). Stands with complex family structures can therefore be expected to represent considerable genetic reservoirs for future selection and adaptation. Forests will only be able to adapt to changing climatic and other environmental conditions if they harbor sufficient genetic diversity to select from. Managed forests already rich in family structures should therefore be managed under the perspective of preserving these structures as much as possible. Low‐complexity stands should be managed to allow development of more complex structures. Stands used for seed production should be as diverse in family structure as possible to generate seed‐lots of high genetic diversity, thereby maximizing the adaptive potential of newly planted stands, especially if these stands are expected to regenerate naturally in the future. The importance of promoting selection and adaptation at the planting stage has already been emphasized by Sork et al. (2002) in the context of restoration of Californian *Q. lobata* née populations. Our results extend their conclusion to also include managed stands to enable selection and adaptation and avoid loss of genetic diversity during recurring production and regeneration cycles.

Our study demonstrates that extensive, apparently homogenous oak forests are far from uniform on the genetic level. On the contrary, they form highly complex mosaics of remarkably small local neighborhoods. This counterbalances the levelling effect of long‐distance dispersal (Felsenstein, 1976) and contributes to the species’ adaptive and evolutionary potential. Incorporating these dynamics into forest management, conservation and restoration can thereby support the conservation of forest genetic diversity by “maintaining the dynamic processes that generate and maintain it” (Savolainen, 2000).

## CONFLICT OF INTEREST

The authors declare no conflict of interest.

## AUTHOR CONTRIBUTION

**Pascal Eusemann:** Conceptualization (equal); Formal analysis (lead); Investigation (lead); Methodology (equal); Project administration (lead); Resources (equal); Validation (equal); Visualization (lead); Writing‐original draft (lead); Writing‐review & editing (equal). **Heike Liesebach:** Conceptualization (equal); Formal analysis (supporting); Funding acquisition (lead); Methodology (equal); Resources (equal); Supervision (lead); Validation (equal); Writing‐review & editing (equal).

## Supporting information

Appendix S1‐S5

## Data Availability

Microsatellite genotypes and sampling locations archived in Dryad DOI: (https://doi.org/10.5061/dryad.3n5tb2rh6).
