# Supplementary material for: Small‐scale genetic structure and mating patterns in an extensive sessile oak forest (Quercus petraea (Matt.) Liebl.)
Source: Ecol Evol. 2021 May 13;11(12):7796–809. doi: 10.1002/ece3.7613 (PMC8216985; doi:10.1002/ece3.7613)
Supplement: Supplementary file 1 — Appendix S1‐S5 [file ECE3-11-7796-s001.docx]

**Appendix**

**Small-scale genetic structure and mating patterns in an extensive sessile oak forest (*Quercus petraea* (Matt.) Liebl.)**

Pascal Eusemann*, Heike Liesebach

*Corresponding author: pascal.eusemann@thuenen.de





**Appendix S1:** Distribution map of *Quercus petraea*. The yellow marker indicates the location of the study site described in this publication.

Source: Caudullo G., Welk E., San-Miguel-Ayanz J. (2017): Chorological maps for the main European woody species. Data in Brief 12, 662-666. https://doi.org/10.1016/j.dib.2017.05.007.

Original map modified to indicate the location of the study site.

The underlying distribution map has been developed by the European Commission Joint Research Centre (partly based on the EUFORGEN map) and released under Creative Commons Attribution 4.0 International (CC-BY 4.0). Link to the license: <https://creativecommons.org/licenses/by/4.0/>

|  | **Parentage Analysis** | **Sibship Analysis** |
| --- | --- | --- |
|  |  |  |
| **Mating System - I** | Female Polygamy | Female Polygamy |
|  | Male Polygamy | Male Polygamy |
|  |  |  |
| **Mating System - II** | With Inbreeding | With Inbreeding |
|  | Without Clones | Without Clones |
|  |  |  |
| **Species** | Monoecious | Monoecious |
|  | Diploid | Diploid |
|  |  |  |
| **Length of Run** | Medium | Medium |
|  |  |  |
| **Analysis Method** | Full-Likelihood | Full-Likelihood |
|  |  |  |
| **Likelihood Precision** | High | High |
|  |  |  |
| **Run Specifications** | Update Allele Frequencies: No | Update Allele Frequencies: No |
|  | Sibship Scaling: Yes | Sibship Scaling: Yes |
|  |  |  |
| **Sibship Prior** | No Prior | No Prior |
|  |  |  |
| **Number of Candidate Parents** | 246 (adult tree generation) | 0 |
|  |  |  |
| **Probability of parent contained in candidate parent sample** |  |  |
| **Mother** | 0.9 | Not applicable |
| **Father** | 0.5 | Not applicable |
|  |  |  |
| **Null Allele Error Rate** | As calculated by CERVUS | As calculated by CERVUS |
| **Mistyping Error Rate** | 0.001 | 0.001 |
| **Allele Frequencies** | Unknown | Unknown |
|  |  |  |

**Appendix S2:** COLONY parameter settings for the parentage and sibship analyses performed in this study.

|  | **S01** | **S02** | **S03** | **S04** | **S05** | **S06** | **S07** | **S08** | **S09** | **S10** | **S11** | **S12** | **S13** | **S14** | **S15** | **S16** | **S17** | **S18** | **S19** | **S20** | **S21** | **S22** | **S23** | **S24** | **S25** |
| --- | --- | --- | --- | --- | --- | --- | --- | --- | --- | --- | --- | --- | --- | --- | --- | --- | --- | --- | --- | --- | --- | --- | --- | --- | --- |
| **S01** |  | 0.123 | 0.073 | 0.156 | 0.151 | 0.126 | 0.100 | 0.120 | 0.154 | 0.105 | 0.095 | 0.182 | 0.085 | 0.101 | 0.109 | 0.136 | 0.238 | 0.060 | 0.142 | 0.058 | 0.109 | 0.098 | 0.084 | 0.099 | 0.104 |
| **S02** | 0.039 |  | 0.124 | 0.168 | 0.123 | 0.131 | 0.147 | 0.199 | 0.143 | 0.134 | 0.123 | 0.199 | 0.116 | 0.168 | 0.127 | 0.118 | 0.246 | 0.105 | 0.203 | 0.097 | 0.186 | 0.158 | 0.122 | 0.117 | 0.108 |
| **S03** | 0.025 | 0.046 |  | 0.020 | 0.100 | 0.097 | 0.063 | 0.124 | 0.127 | 0.139 | 0.143 | 0.190 | 0.082 | 0.113 | 0.093 | 0.079 | 0.191 | 0.075 | 0.148 | 0.098 | 0.109 | 0.076 | 0.069 | 0.102 | 0.091 |
| **S04** | 0.061 | 0.070 | 0.008 |  | 0.146 | 0.147 | 0.121 | 0.176 | 0.185 | 0.184 | 0.176 | 0.236 | 0.136 | 0.184 | 0.183 | 0.152 | 0.270 | 0.149 | 0.216 | 0.164 | 0.169 | 0.157 | 0.102 | 0.150 | 0.141 |
| **S05** | 0.053 | 0.046 | 0.041 | 0.066 |  | 0.075 | 0.071 | 0.146 | 0.114 | 0.109 | 0.135 | 0.169 | 0.112 | 0.156 | 0.121 | 0.123 | 0.246 | 0.075 | 0.127 | 0.068 | 0.100 | 0.110 | 0.138 | 0.112 | 0.143 |
| **S06** | 0.045 | 0.050 | 0.041 | 0.067 | 0.032 |  | 0.045 | 0.102 | 0.128 | 0.090 | 0.107 | 0.130 | 0.040 | 0.117 | 0.114 | 0.055 | 0.228 | 0.053 | 0.102 | 0.059 | 0.118 | 0.083 | 0.108 | 0.069 | 0.099 |
| **S07** | 0.037 | 0.058 | 0.027 | 0.058 | 0.031 | 0.020 |  | 0.088 | 0.132 | 0.122 | 0.119 | 0.150 | 0.062 | 0.094 | 0.101 | 0.099 | 0.226 | 0.051 | 0.081 | 0.070 | 0.114 | 0.091 | 0.098 | 0.090 | 0.113 |
| **S08** | 0.042 | 0.072 | 0.050 | 0.079 | 0.059 | 0.043 | 0.038 |  | 0.165 | 0.089 | 0.095 | 0.160 | 0.058 | 0.081 | 0.142 | 0.122 | 0.218 | 0.084 | 0.079 | 0.102 | 0.127 | 0.124 | 0.150 | 0.086 | 0.125 |
| **S09** | 0.049 | 0.049 | 0.047 | 0.076 | 0.043 | 0.049 | 0.052 | 0.061 |  | 0.070 | 0.116 | 0.134 | 0.116 | 0.175 | 0.174 | 0.116 | 0.231 | 0.128 | 0.154 | 0.083 | 0.178 | 0.146 | 0.119 | 0.123 | 0.077 |
| **S10** | 0.035 | 0.048 | 0.054 | 0.078 | 0.043 | 0.036 | 0.050 | 0.035 | 0.026 |  | 0.000 | 0.129 | 0.068 | 0.141 | 0.133 | 0.091 | 0.240 | 0.085 | 0.125 | 0.038 | 0.174 | 0.109 | 0.125 | 0.089 | 0.091 |
| **S11** | 0.032 | 0.044 | 0.055 | 0.075 | 0.052 | 0.042 | 0.049 | 0.037 | 0.041 | 0.000 |  | 0.152 | 0.054 | 0.115 | 0.136 | 0.095 | 0.216 | 0.075 | 0.115 | 0.051 | 0.164 | 0.111 | 0.132 | 0.090 | 0.094 |
| **S12** | 0.065 | 0.075 | 0.078 | 0.106 | 0.070 | 0.055 | 0.065 | 0.066 | 0.051 | 0.052 | 0.060 |  | 0.098 | 0.194 | 0.203 | 0.163 | 0.253 | 0.114 | 0.163 | 0.099 | 0.200 | 0.163 | 0.135 | 0.147 | 0.143 |
| **S13** | 0.028 | 0.040 | 0.031 | 0.058 | 0.043 | 0.016 | 0.025 | 0.022 | 0.041 | 0.025 | 0.020 | 0.038 |  | 0.061 | 0.097 | 0.047 | 0.160 | 0.019 | 0.053 | 0.036 | 0.092 | 0.072 | 0.072 | 0.039 | 0.038 |
| **S14** | 0.033 | 0.057 | 0.043 | 0.076 | 0.058 | 0.045 | 0.038 | 0.031 | 0.059 | 0.051 | 0.042 | 0.073 | 0.022 |  | 0.153 | 0.134 | 0.168 | 0.106 | 0.104 | 0.110 | 0.153 | 0.115 | 0.136 | 0.116 | 0.111 |
| **S15** | 0.041 | 0.050 | 0.040 | 0.085 | 0.052 | 0.050 | 0.046 | 0.061 | 0.068 | 0.055 | 0.055 | 0.087 | 0.039 | 0.060 |  | 0.093 | 0.246 | 0.069 | 0.153 | 0.121 | 0.136 | 0.124 | 0.106 | 0.105 | 0.127 |
| **S16** | 0.047 | 0.044 | 0.032 | 0.068 | 0.050 | 0.023 | 0.043 | 0.049 | 0.043 | 0.035 | 0.037 | 0.067 | 0.018 | 0.050 | 0.040 |  | 0.144 | 0.077 | 0.125 | 0.075 | 0.132 | 0.074 | 0.111 | 0.080 | 0.053 |
| **S17** | 0.083 | 0.090 | 0.077 | 0.119 | 0.098 | 0.092 | 0.096 | 0.088 | 0.085 | 0.092 | 0.083 | 0.103 | 0.061 | 0.064 | 0.103 | 0.059 |  | 0.232 | 0.258 | 0.186 | 0.219 | 0.248 | 0.237 | 0.183 | 0.132 |
| **S18** | 0.021 | 0.038 | 0.030 | 0.065 | 0.030 | 0.022 | 0.022 | 0.034 | 0.046 | 0.033 | 0.029 | 0.046 | 0.007 | 0.039 | 0.030 | 0.031 | 0.090 |  | 0.049 | 0.048 | 0.095 | 0.076 | 0.048 | 0.050 | 0.080 |
| **S19** | 0.045 | 0.068 | 0.055 | 0.088 | 0.047 | 0.039 | 0.032 | 0.029 | 0.052 | 0.044 | 0.041 | 0.062 | 0.018 | 0.036 | 0.060 | 0.046 | 0.095 | 0.018 |  | 0.069 | 0.110 | 0.100 | 0.143 | 0.104 | 0.129 |
| **S20** | 0.018 | 0.032 | 0.036 | 0.066 | 0.025 | 0.023 | 0.027 | 0.037 | 0.028 | 0.013 | 0.018 | 0.037 | 0.012 | 0.037 | 0.046 | 0.028 | 0.067 | 0.018 | 0.023 |  | 0.074 | 0.077 | 0.093 | 0.075 | 0.069 |
| **S21** | 0.036 | 0.065 | 0.042 | 0.073 | 0.039 | 0.046 | 0.046 | 0.049 | 0.063 | 0.063 | 0.060 | 0.078 | 0.032 | 0.054 | 0.055 | 0.050 | 0.085 | 0.035 | 0.038 | 0.024 |  | 0.076 | 0.141 | 0.099 | 0.154 |
| **S22** | 0.034 | 0.057 | 0.030 | 0.069 | 0.044 | 0.034 | 0.038 | 0.049 | 0.053 | 0.042 | 0.042 | 0.066 | 0.027 | 0.043 | 0.052 | 0.030 | 0.097 | 0.030 | 0.036 | 0.028 | 0.028 |  | 0.104 | 0.104 | 0.108 |
| **S23** | 0.029 | 0.044 | 0.027 | 0.046 | 0.055 | 0.044 | 0.041 | 0.059 | 0.043 | 0.047 | 0.050 | 0.055 | 0.027 | 0.050 | 0.045 | 0.044 | 0.093 | 0.019 | 0.052 | 0.033 | 0.053 | 0.040 |  | 0.102 | 0.088 |
| **S24** | 0.034 | 0.043 | 0.041 | 0.066 | 0.045 | 0.028 | 0.038 | 0.035 | 0.045 | 0.034 | 0.034 | 0.060 | 0.014 | 0.043 | 0.044 | 0.032 | 0.073 | 0.020 | 0.038 | 0.027 | 0.037 | 0.040 | 0.040 |  | 0.065 |
| **S25** | 0.034 | 0.037 | 0.034 | 0.059 | 0.053 | 0.038 | 0.045 | 0.047 | 0.027 | 0.033 | 0.034 | 0.054 | 0.013 | 0.039 | 0.050 | 0.021 | 0.050 | 0.029 | 0.044 | 0.023 | 0.054 | 0.039 | 0.032 | 0.024 |  |

**Appendix S3:** Genetic differentiation between seedling sampling areas. S01 - S25: Seedling sampling area ID. D_EST_-values above the diagonal, F_ST_-values below the diagonal.

**Appendix S4:** Family structure within the adult generation. X- and Y-axes give tree coordinates in meters. Each color represents one family of individuals with first and second degree of relatedness. Due to the large number of small families, only families with at least five members are shown in order to increase clarity and legibility of the figure. Unfilled circles symbolize trees in families of less than five members. The pronounced clustering of individuals visualized in this figure is also represented in the significant positive spatial autocorrelation over small distances found by the SGS analysis.

**Appendix S5:** Family structure within the seedling sampling areas. Each color/pattern represents an individual parent tree. Each pie graph represents one sampling area. The graphs are ordered to reflect the spatial arrangement of the sampling areas within the study site. The proportion of each color/pattern within a pie graph represents the proportion of offspring of this parent tree within the sampling area. To increase clarity, all singletons, that is, parents that contributed only one seedling within the sampling area, are merged within the light grey proportion of each pie graph.
